# Jejunojejunal Intussusception as the Initial Presentation of Non-Hodgkin's B-Cell Lymphoma in an Adult Patient: A Case Report and Review of the Literature

**DOI:** 10.1155/2013/619031

**Published:** 2013-06-17

**Authors:** V. Stohlner, N. A. Chatzizacharias, M. Parthasarathy, T. Groot-Wassink

**Affiliations:** Department of Surgery, Ipswich Hospital, Ipswich, Suffolk IP4 5PD, UK

## Abstract

*Introduction*. Intussusception is a rare cause of bowel obstruction in adults and is usually associated with an underlying pathology, benign, or malignant. This is a report of a case of jejunojejunal intussusception secondary to non-Hodgkin's B-cell lymphoma in an adult patient. *Case Presentation*. A 74-year-old male with no previous significant medical history presented with symptoms of acute intestinal obstruction. A CT scan of the abdomen and pelvis revealed 2 areas of jejunojejunal intussusception, which were surgically managed successfully. Histopathological examination of the specimen revealed the presence of high grade diffuse large B-cell-type non-Hodgkin's lymphoma, and the patient was referred to the oncology team for further management. *Discussion*. B-cell lymphoma is a rare but well-documented cause of intussusception in adults, with most cases being at the ileocolic region. We present a rare case of jejunojejunal intussusception as the initial presentation of non-Hodgkin's B-cell lymphoma in an adult patient.

## 1. Introduction

Intussusception of the bowel, first reported in 1674, can be described as the telescoping of a proximal segment of the intestine within the lumen of the adjacent segment [1]. It is considered a rare cause of bowel obstruction in adults with approximately 95% of the total number of cases of intussusception seen in children. A small number of cases seen in adults are idiopathic, with no lead point lesion identified. However, the majority of cases are secondary to pathology, such as carcinoma, polyps, diverticulum, or other lesions [1].

Lymphoma is a very rare cause of adult intussusception, with only 36 cases reported in the literature between 2000 and 2011 [2]. Of these 11 were due to the non-Hodgkin's B-cell type. 

We report a rare case of non-Hodgkin's B-cell lymphoma presenting with 2 areas of jejunojejunal intussusception. A similar presentation, with one area of jejunojejunal intussusception, has been reported only once previously [3].

## 2. Case Presentation

The patient was a 74-year-old male who presented to the emergency department of our hospital complaining of severe abdominal pain associated with vomiting and failure to defecate for three days. A detailed history revealed that his symptomatology included a 2 month history of significant weight loss (12 Kg over 2-months) and poor appetite. He also complained of a recent change in bowel habit, alternating between constipation and loose motions, and an intermittent colicky abdominal pain, which was relieved by passing flatus and defecation.

Clinical examination revealed a distended, soft abdomen, with generalised tenderness, but no peritonism, organomegaly, or masses. Bowel sounds were reduced and rectal examination was unremarkable. All other aspects of the examination were normal. Initial blood investigations showed raised inflammatory markers (CRP 231 mg/L, white blood cells 17.1 × 10^9^/L) and anaemia (haemoglobin 9.5 g/dL, MCV 71 fL). An abdominal X-ray showed distended loops of small bowel. 

**Figure 1:**
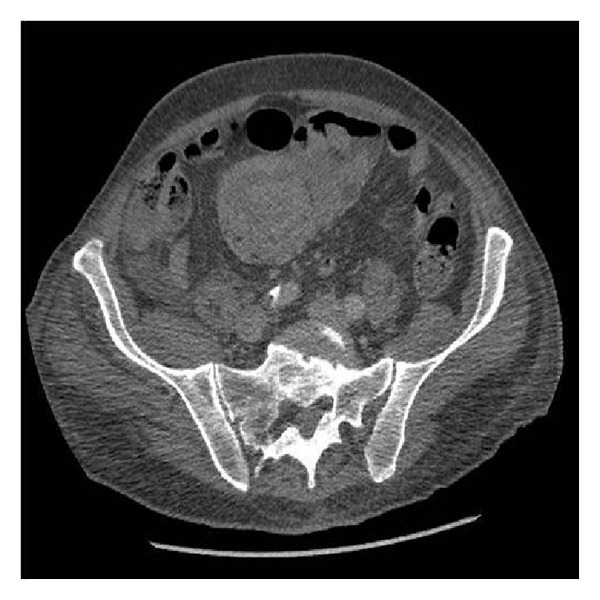
CT scan illustrating the area of intussusception.

An initial diagnosis of bowel obstruction was made and the patient was admitted for conservative management. Further investigations with a computer tomography (CT) scan of the abdomen and pelvis with contrast showed a 9 cm circumferential wall thickening of the small bowel inferior to the transverse colon, associated with two areas of intussusception and further abnormal loops of bowel (Figure 1). A decision for an emergency laparotomy was reached. Intraoperative findings confirmed two separate areas of jejunojejunal intussusception. The involved parts of the bowel were resected and an end-to-end anastomosis was performed. Furthermore, during inspection of the bowel numerous thick stalked polyps were identified within the jejunum. The seven largest of these were resected by separate enterotomies. 

Postoperatively the patient was managed on the Intensive Care Unit and was discharged to the ward on day 6. His course was complicated with an upper gastrointestinal bleed on day 7 due to a Mallory-Weis tear, which was managed with an emergency esophagogastroscopy. The remaining of the recovery period was uncomplicated and the patient was discharged after a total of 26 days of hospital stay. 

**Figure 2:**
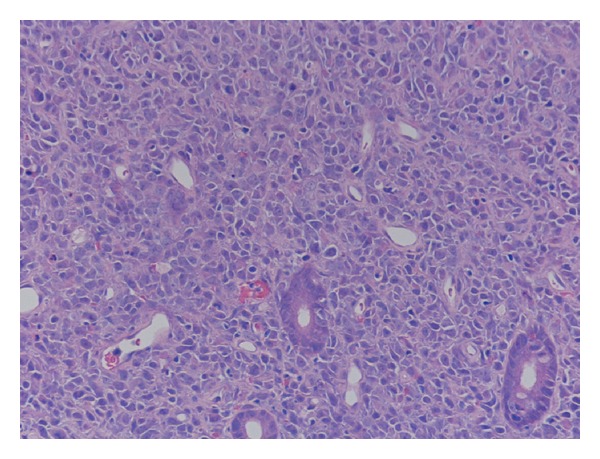
Hematoxylin and eosin stain showing intestinal glands surrounded by large pleomorphic lymphoid cells (magnification ×200).

The histopathological examination of the specimens revealed features of high grade non-Hodgkin's B-cell lymphoma of the diffuse large B-cell type (Figure 2). Therefore the patient was referred to the oncology team for appropriate further management. 

## 3. Discussion

Primary lymphoma of the gastrointestinal tract accounts for 30–40% of lymphomas arising extranodally and comprises 10–15% of all non-Hodgkin lymphomas [4]. Symptomatology varies and can include any combination of the following: dyspepsia, epigastric pain, abdominal pain, nausea, vomiting, diarrhoea, weight loss, malabsorption, obstruction, anaemia, and to a lesser extent ulceration, perforation, and intussusceptions [5–7]. In the Western population, 60% to 80% of intestinal lymphomas are B-cell lymphomas, mostly of the diffuse type. Most commonly they are derived from the B cells in the lymphoid tissue of the lamina propria and submucosa of the ileum, where the greatest concentration of gut-associated lymphoid tissue is located [6, 7].

Intussusception is rarely considered clinically in the differential diagnosis of adult patients with vague abdominal complaints. Therefore diagnosis is usually made on CT or during exploratory laparoscopy/laparotomy [8]. The clinical importance of intussusception in adults is that it is usually due to an underlying pathology. Therefore, surgical resection, with adequate margins in case of suspected malignancy, is considered as the definitive management in this population [2, 3, 9]. Although rare, intussusception is a recognised presenting feature of lymphoma [2, 5–7, 9]. The most common recognised site is the ileocolic region [2, 5–9]. We report a case of non-Hodgkin's B-cell lymphoma presenting with 2 areas of jejunojejunal intussusception. This is the first case with more than one area of small intestinal intussusception due to lymphoma in the published literature, with one case of a single intussusception been also described [3]. These two cases demonstrate that lymphoma polyps in any part of the small bowel can cause intussusception. Excision of such polyps with critical size at the time of surgery appears advisable. Urgent chemotherapy is required to prevent enlargement of other smaller lesions.

In conclusion, intussusception in the adult population is usually associated with an underlying cause, including lymphoma. This report illustrates a rare case of a jejunojejunal intussusception secondary to a high grade diffuse large B-cell-type non-Hodgkin's lymphoma.
